# Detection of Lipid Oxidation in Infant Formulas: Application of Infrared Spectroscopy to Complex Food Systems

**DOI:** 10.3390/foods9101432

**Published:** 2020-10-09

**Authors:** Samar Daoud, Elias Bou-Maroun, Gustav Waschatko, Benjamin Horemans, Renaud Mestdagh, Nils Billecke, Philippe Cayot

**Affiliations:** 1 Unité Mixte “Procédés Alimentaires et Microbiologiques”, Université Bourgogne Franche-Comté, AgroSup Dijon, PAM UMR A 02.102, F-21000 Dijon, France; elias.bou-maroun@agrosupdijon.fr (E.B.-M.); philippe.cayot@agrosupdijon.fr (P.C.); 2 Cargill R&D Centre Europe BVBA Havenstraat 84, B-1800 Vilvoorde, Belgium; gustav_waschatko@cargill.com (G.W.); benjamin_horemans@cargill.com (B.H.); renaud_mestdagh@cargill.com (R.M.); nils_billecke@cargill.com (N.B.)

**Keywords:** infant formula, lipid oxidation, infrared spectroscopy, NIR, ATR-FTIR, DHA

## Abstract

Fish- or algal oils have become a common component of infant formula products for their high docosahexaenoic acid (DHA) content. DHA is widely recognized to contribute to the normal development of the infant, and the European Commission recently regulated the DHA content in infant formulas. For many manufacturers of first-age early life nutrition products, a higher inclusion level of DHA poses various challenges. Long-chain polyunsaturated fatty acids (LC-PUFAs) such as DHA are very prone to oxidation, which can alter the organoleptic property and nutritional value of the final product. Traditional methods for the assessment of oxidation in complex systems require solvent extraction of the included fat, which can involve harmful reagents and may alter the oxidation status of the system. A rapid, efficient, non-toxic real-time method to monitor lipid oxidation in complex systems such as infant formula emulsions would be desirable. In this study, infrared spectroscopy was therefore chosen to monitor iron-induced oxidation in liquid infant formula, with conjugated dienes and headspace volatiles measured with GC-MS as reference methods. Infrared spectra of infant formula were recorded directly in mid- and near-infrared regions using attenuated total reflectance Fourier-transform (ATR-FTIR) and near-infrared (NIRS) spectrophotometers. Overall, good correlation coefficients (R^2^ > 0.9) were acquired between volatiles content and infrared spectroscopy. Despite the complex composition of infant formula containing proteins and sugars, infrared spectroscopy was still able to detect spectral changes unique to lipid oxidation. By comparison, near-infrared spectroscopy (NIRS) presented better results than ATR-FTIR: prediction error ATR-FTIR 18% > prediction error NIRS 9%. Consequently, NIRS demonstrates great potential to be adopted as an in-line or on-line, non-destructive, and sustainable method for dairy and especially infant formula manufacturers.

## 1. Introduction

Omega-3 long-chain polyunsaturated fatty acids (LC-PUFAs) such as alpha-linolenic acid (ALA, C18:3), eicosapentaenoic acid (EPA, C20:5), and docosahexaenoic acid (DHA, C22:6) are well known to improve brain and nervous system development in infants and young children [1,2,3,4]. The European Food Safety Authority (EFSA), among other authoritative bodies, recommends a higher intake of omega-3 fatty acids, especially at a young age [3]. In the European Union, the addition of DHA to infant formula and follow-on formula became compulsory [5]. Infant milk formulas represent the only food for newborns, performing a crucial nutritional role [6]. Formulas enriched in DHA are extremely prone to oxidation, which poses the risk of exposing the infant to harmful degradation products. Not only do the included LC-PUFAs have a high degree of unsaturation [7], but the addition of prooxidants such as iron to meet nutritional requirements [5] also poses a challenge to the stability of the final product. Hydroperoxides are the first oxidation products and are very reactive, breaking down rapidly to aldehydes, ketones, and other volatiles. These secondary products of lipid oxidation are responsible for rancidity and off-flavors [8]. Oxidized flavors are noticeable in stored milk at low levels of oxidation (peroxide value <1 meq.O_2_/kg oil). “Fishy” and “metallic” attributes were correlated with increased concentrations of hexanal and heptanal and have been shown to be particularly evident in infant milk formula enriched with LC-PUFA [8,9,10]. Moreover, multiple lipid oxidation products (such as free radicals, reactive oxygen species, and aldehydes) may exert detrimental effects on vital biological processes, potentially leading to tissue injuries and increasing the risk for degenerative diseases [11,12,13].

To ensure the safety and quality of the supplemented food, an efficient method to measure lipid oxidation is required. So far, the determination of hydroperoxides (peroxide value (PV) or conjugated diene (CD) value) and aldehydes (thiobarbituric acid reactive species (TBARS)) in infant formula were conventionally used [9,14,15]. However, determining the oxidative status of oil in food matrices such as infant formula requires the extraction of fats and oils. This step is time consuming, involves harmful solvents, and may induce under- or overestimation of oxidation levels [15,16]. In addition, in-line implementation in industrial production lines is not possible. Liquid or gas chromatography was also used traditionally to monitor lipid oxidation in infant formulas [10,17,18]. However, chromatography analysis requires long sample preparation and the addition of reagents and standards for quantification. A rapid, accurate, and direct method may reduce effort, analysis time, and, subsequently, costs.

Spectral and multivariate analyses enable the use of infrared spectroscopy as a practical tool for food examination [19]. In the mid infrared region (4000–600 cm^−1^), spectral signatures result from the fundamental stretching, bending, and rotating vibrations of the tested sample molecules. Absorptions in near-infrared (NIR) regions 800 to 2500 nm (12,500–4000 cm^−1^) correspond to overtones and combinations of the fundamental bands [20]. Infrared spectroscopy has been increasingly applied for several food matrices to measure adulteration in olive oil [21,22], water content [23], fat, and protein content in cereal food [24]. However, infrared spectroscopy is also very useful to measure lipid oxidation. Good correlations were observed between spectral data and the formation of oxidation products. Several studies presented near infrared spectroscopy (NIRS) correlations with oxidation products of bulk oils [25,26,27]. Spectra in the mid-infrared region using FTIR also showed good prediction of oxidation levels in bulk oil [28,29,30,31].

However, studies using infrared spectroscopy (NIRS and FTIR) to monitor lipid oxidation in complex food systems are still scarce, and only a few studies analyzed oil oxidation in emulsions [32,33,34,35,36,37]). These emulsion–oxidation studies depend on a fat extraction step prior to analysis. NIRS applications in the oxidation monitoring of complex “foods” and not bulk oils were limited: whole walnut kernels [38], cereal food products [39], and potato crisps [40]. Infrared spectroscopy was also shown once to monitor oxidation in infant formula powder [41]. However, in these applications, infrared spectroscopy was applied after fat extraction and on products with low water contents.

Infrared spectroscopy offers several advantages over conventional methods, including high speed, minimal sample volume and preparation, and no chemical wastes. In addition, compared to other spectroscopic tools (e.g., attenuated total reflectance Fourier-transform infrared ATR-FTIR, or Raman) NIRS sensors can be used in-line or on-line for the liquid process streams. Regarding all these criteria, infrared spectroscopy is considered, as a “green analytical tool” [20,42,43]. Its use for lipid oxidation evaluation could be a practical approach for food industries where quick, reliable, and accurate assessment of the quality of products are needed.

This study aims to evaluate the ability of infrared spectroscopy in near- (NIRS) and mid- (ATR-FTIR) infrared regions to predict iron-initiated lipid oxidation in infant milk formulas without prior fat extraction. Milk formulas were prepared and kept at different storage conditions. Iron was used as an oxidation initiator, and a blend of antioxidants was included in the formula. Oxidation levels were monitored as well by standard technics such as conjugated diene values (CD), and total volatile content measured by solid-phase microextraction (SPME) gas-chromatography coupled to mass spectrometry (GC-MS). Correlations of these standard values with spectral changes were performed using partial least squares regression (PLSR).

## 2. Materials and Methods

Skimmed milk powder LF100, whey protein (Lactarmor™ DM 90), lactose, and glucose were obtained from Armor Proteins, Rennes, France. The fat blend was a mixture of fish and vegetables oils (45% (*v*/*v*) palm, 20% (*v*/*v*) coconut, 25% (*v*/*v*) rapeseed, and 10% (*v*/*v*) sunflower oils) commonly used in the making of an infant formula. This mixture was delivered by Cargill R&D center (Vilvoorde, Belgium). The mixture contained 1.3% *w*/*w* of DHA in the final product. This percentage corresponds to the recent regulations of DHA addition in infant formula [5]. The antioxidants were ascorbyl palmitate (CAS Number 137–66–6), citric acid monohydrate (CAS Number 5949–29–1), and soybean lecithin, which was supplied by Cargill R&D Europe Center (Vilvoorde, Belgium).

Ferrous sulfate heptahydrate (CAS Number 7782–63–0) was added to initiate lipid oxidation. Fat extraction and conjugated diene measurements were performed using isooctane (CAS Number 540–84–1), 2-propanol (CAS Number 67–63–0), and sodium chloride (CAS Number 7647–14–5). All reagents and solvents were analytical grade and purchased from Merck Sigma Aldrich (Munich, Germany).

### 2.1. Infant Milk Formula Production and Oxidation Conditions

Infant milk formulas consisted of 50% (*w*/*w*) of a powder mixture and 50% (*w*/*w*) of water containing ferrous sulfate added at 195 ppm (final concentration in formulas 3.5 mM of Fe^2+^). First, standardized tap water (300 g) was preheated at 60 °C with stirring at 800–1000 rpm. Second, skimmed milk powder (40.8 g) and whey protein (112.5 g) were added. After 5 min of continuous stirring, lactose (47.7 g) and glucose (14.1 g) were poured in and stirred for 30 min (500 rpm) at 60 °C. Oil mixture (84.9 mg) containing fish oil was added to the aqueous phase to obtain a final volume fraction of 14% (*v*/*v*). A pre-emulsification step was completed using a homogenizer (Ultra Turrax^®^ T25, IKA^®^ Labortechnik, Staufen, Germany) for 30 s at 12,000 rpm. Samples were then homogenized at 60 °C with a two-stage valve-type homogenizer (220/30 bar) for three passages (Panda plus, GEA, Niro Soavi, Parma, Italy). The pH was adjusted to 6.5–6.7 with a 1 M potassium hydroxide (KOH) solution. Samples were heated for 1 min at 85 °C and stirred at 400 rpm to mimic the pasteurization step. Using this protocol, two types of infant milk formulas were produced. The first recipe contained no antioxidants (No-AOx). For the second recipe, a mixture of antioxidants containing ascorbyl palmitate (250 ppm) and citric acid (235 ppm) was included. In addition, soybean lecithin (1900 ppm) was added as an emulsifier and potential antioxidant (AOx).

Four grams of each sample were transferred into 20 mL brown capped vials. These samples were flushed with oxygen for 30 s and kept at 50, 60, and 70 °C. These conditions were set to significantly increase the oxidation rate. At lower temperatures (<40 °C) and in sealed containers, infant milk formula can remain stable for at least 3 months [9,18]. For each set of samples, three replicates were prepared. Through storage, samples were analyzed with infrared spectroscopy and reference methods.

### 2.2. Conjugated Diene Determination (CD Values)

The determination of CD values was selected in this study as a reference method. First, the fat phase was extracted as described in our previous work [32]. Briefly, samples (0.2 g) isooctane/2-propanol (3/1, *v*/*v*) solution (1 mL) and sodium chloride (0.024 g) were placed in plastic tubes. The mixture was strongly shaken using a vortex device for 1 min and left to stand for 1 min for phase separation.

Afterward, the American oil chemist society (AOCS) official method Ti 1a–64 [44] was applied with slight modifications. The upper layer (20–50 µL) was diluted in isooctane. The UV–Vis spectra were recorded in the range of 200 to 300 nm using a UV–Vis spectrophotometer (SAFAS, Monaco). The data interval and the band path were set at 1 and 2 nm, respectively. The conjugated dienes have a maximum absorbance near 233 nm. Pure isooctane was used as a reference to set the spectrometer to zero absorbance.

### 2.3. Total Volatile Measurements using GC-MS

Volatiles were extracted by headspace solid-phase microextraction (HS-SPME) using MPS autosampler (Gerstel, Baltimore, MD, USA). Prior to fiber insertion, 4 g of infant formula in a 20 mL vial was placed at 70 °C for 2 min at an agitation rate of 500 rpm. A (1 cm 100 μm) polydimethylsiloxane (PDMS) fiber (Supelco, Bellefonte, PA, USA) was then introduced into the HS and the vial was agitated at 250 rpm for 5 min at 70 °C.

Afterward, volatiles were thermally desorbed into the injector of an Agilent 7890 gas chromatograph coupled to a time-of-flight accurate mass spectrometer (GC/Q-TOF-MS, Agilent technologies, Santa Clara, CA, USA). The injections were splitless for 1 min at 300 °C. A HP-5MS column (30 m × 250 µm i.d. × 0.25 µm film thickness) from Agilent Technologies Inc. (Santa Clara, CA, USA) was used. The column temperature was programmed as follows: initial hold for 2 min at 40 °C, followed by a 15 °C/min ramp to 185 °C and then, 120 °C/min ramp to 300 °C, 1 min hold. The carrier gas was helium (flow rate of 1.5 mL/min). The detector temperature was placed at 300 °C. The TOF-MS was operated in electron impact mode (ionization energy of 70 eV). All samples were analyzed, at least, in triplicates to measure the volatiles derived from lipid oxidation. Results were expressed as area responses (counts).

### 2.4. Near- and Mid-Infrared Measurements

The infrared spectrometer Nicolet iS50-FT-IR, from Thermo Scientific (Waltham, MA, USA), was used to record infrared spectra. The spectrometer was coupled to an ATR accessory (ZnSe crystal, Smart iTX, Thermo Scientific, USA). Samples were spread directly on the crystal surface, and the absorbance spectrum was collected. A dry and empty ATR cell was used as a reference. The ATR spectra were averaged over 16 scans from 4000 to 600 cm^−1^ and recorded with a resolution of 4 cm^−1^. All measurements were conducted at room temperature. The crystal was cleaned with ethanol and water.

For the near-infrared region (NIR), a near-IR integrating sphere (Thermos Scientific, Waltham, MA, USA) accessory was coupled to the spectrometer. Likewise, spectra were measured directly with no sample preparation. The spectrum was recorded in the range of 10,000–4000 cm^−1^, with 16 scans and 8 cm^−1^ resolution. Finally, measurements were made in triplicates.

### 2.5. Pretreatment and Multivariate Data Analysis

Data analysis was performed using the TQ Analyst software (version 9; Thermo Scientific, Waltham, MA, USA). PLSR was used to establish correlations between the infrared spectra features and the oxidation level obtained by the standard reference methods (conjugated diene and volatile contents by GC-MS).

Background noises were observed in the spectral data. Thus, preprocessing the spectral data was essential to produce accurate and stable calibration models [19]. Various methods were investigated. In the near-infrared region, the second derivative (gap 23 points, segment 19 points) was chosen to remove baseline shifts and increase the spectral resolution [45]. PLSR models were designed using the spectral range of 800–2400 nm for log 1/T data. Conversely, the range from 4000–600 cm^−1^ was selected for the mid-infrared region. In this region, the standard normal variate (SNV) followed by baseline correction was used to reduce interference and baseline shifts and to increase spectral resolution.

Afterward, samples were divided randomly into two sets for calibration and validation purposes, using the random feature provided in the TQ Analyst software. Since the validation of this model was ensured by an external set of samples (validation set), the root mean square error of validation (RMSEV) can be defined as the RMSE of prediction (RMSEP). The number of PLSR factors was selected to ensure a minimum root mean square error of prediction (RMSEP) [46]. The coefficient of determination (R^2^), the root mean square error of calibration (RMSEC), and prediction (RMSEP) were selected to evaluate the performance of each model. When the coefficient of determination (R^2^) was closer to 1, and the RMSEC and RMSEP were the lowest, a model was judged efficient [47,48,49]. Furthermore, the ratio of the standard error of performance (SEP) to the standard error of calibration (SEC) must be less than 1.2. However, to identify the usage of the developed model, the ratio of the data range (R) to the SEP should be evaluated. When it is superior or equal to 4, the model can only be used for screening calibration. When this ratio is superior or equal to 10, the model is suitable for quality control. Once this ratio is superior or equal to 15, the model can be applied for quantification studies [50]. The residual prediction deviation (RPD) value can be also checked for model performance. The RPD is calculated as the ratio of (standard error of) performance to (standard) deviation (RPD) = SD of data/SEP. SD/SEP ≥ 2.5 means screening in breeding programs; SD/SEP ≥ 5 means acceptable for quality control; and SD/SEP ≥ 8 means good for process control, development, and applied research [50].

To assess significant differences, the analysis of variance (one-way ANOVA), followed by means comparison using the Tukey test, was used. The level of significance was set to 95% (*p* = 0.05). Statistical tests were performed using the Minitab^®^ (version 18.1; State College, PA, USA).

## 3. Results and Discussion

### 3.1. Oxidation Monitoring by Reference Methods

#### 3.1.1. Determination of Conjugated Diene Values

**Figure 1:**
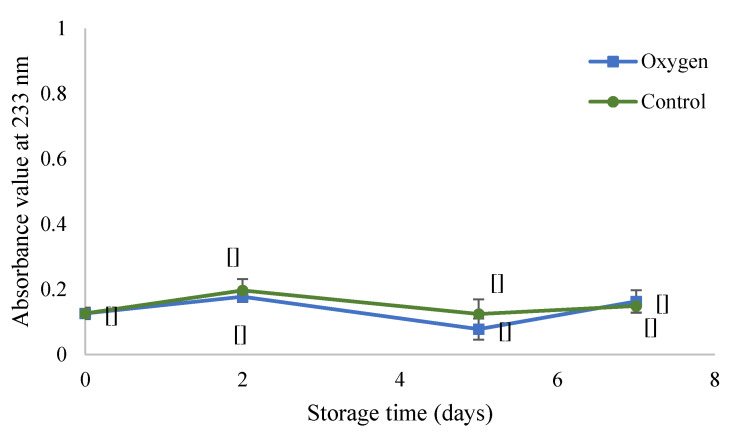
Variation in absorbance at 233 nm (conjugated diene (CD) values) of liquid infant milk formula stored under pure oxygen in the head space (■ oxygen) or under air (●).

The oxidation level was evaluated using the conjugated dienes method. Figure 1 shows the variation of absorbance at 233 nm for infant milk formulas (with iron and without antioxidants addition) stored in the dark at 70 °C for 7 days. The value of the absorbance at 233 nm was extremely low and below the linearity range (0.2–0.8) of the UV spectrometer. However, signs of lipid oxidation (off-flavors, color changing) for both conditions were detected.

Iron (Fe^2+^) was added at 3.5 10^3^ µM. Formulas were flushed with oxygen (**■**) or not (**●**) (control sample under normal air atmosphere). Samples were kept in the dark for 7 days at 70 °C. Data are shown as means of two replicates and three repetitions each one ± SD (*n* = 6). Common letters indicate no significant difference within the same conditions according to ANOVA (*p* < 0.05).

The CD value was around 1 mmol of eq. peroxide/kg emulsion for both conditions (oxygen or ambient air), without any differences. Determination of CD was unsuitable for detecting the lipid oxidation in infant milk formula in this study. When monitoring conjugated dienes, relatively long storage intervals i.e., 3 to 6 months, were necessary to notice changes in the stability of infant formulas [10]. In our previous work on the oil-in-water emulsion, the CD method allowed the monitoring of lipid oxidation [32], which was due to the higher content of sensitive oils in the samples there, emulsion contained 20% (*v*/*v*) pure tuna oil. In contrast, infant milk formula contains 14% (*v*/*v*) of fat which represents a mixture of fish (approx.1.3% (*v*/*v*) of total fat) and vegetable oils. The formation of conjugated dienes is closely related to the number of unsaturation of fatty acids [11]. Therefore, in infant milk, the content of conjugated dienes is not representative of the oxidation level. Subsequently, oxidation was assessed using GC-MS as a reference method.

#### 3.1.2. Measurement of Released Volatiles by HS-SPME/GC-MS

HS-SPME/GC-MS was used to monitor changes in the key volatile compounds in infant milk formula. The lipid profile of infant formula differs regarding the intended uses (first age, follow-on formulas, etc.). Nevertheless, the main fat part of all infant formulas consists of a mixture of vegetable oils (coconut, palm, corn, sunflower, rapeseed, etc.). Palmitic (C16:0), oleic (C18:1), linoleic (C18:2), and linolenic (C18:3) acids are the major fatty acids [51]. Once oxidized, these fatty acids will be transformed into hydroperoxides (LOOH). The decomposition of LOOH by scission reaction leads to lower-molecular-weight compounds such as aldehydes, ketones, alkanes, and alcohols (secondary products of oxidation). These volatiles are responsible for off-flavors and rancidity [11].

Ten aldehydes were chosen to represent oxidation products of oleic, linoleic, and linolenic acids. Octanal, nonanal, decanal, and 2-decenal were used to track oleic acid oxidation. Hexanal, 1-octene-3-ol, 2-nonenal, and 2,4-decadienal were used as key volatiles of linoleic acid oxidation. As for linolenic acid, 2-pentenal/2-hexenal was measured as the main volatile of its oxidation. These are the main aldehydes produced for each fatty acid [10,52]. Hexanal was the major aldehyde with the highest response area value (Supplementary Materials Figure S1). The other compounds slightly increased. However, 2,4-decadienal showed a small decrease (Supplementary Materials Figure S2). This can be explained by the transformation of 2,4-decadienal in 2-octenal and hexanal by retro-aldolization. This mechanism was described in a study on poultry meat [53]. Therefore, the sum of all the response areas for the ten aldehydes was used to interpret the results.

**Figure 2:**
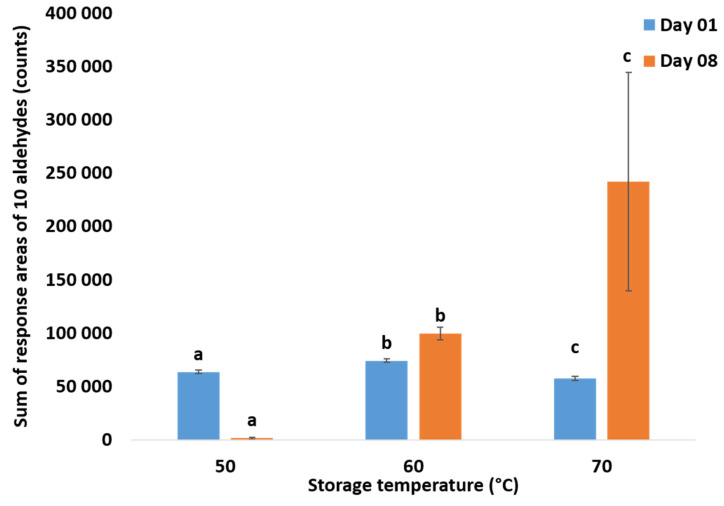
Impact of storage temperature on the sum of volatiles (response areas) determined by solid-phase microextraction (SPME)-GC-MS in a liquid infant milk formula. Samples were stored in the dark for 8 days at 50, 60, and 70 °C and contained no antioxidants. Values are presented as means. Error bars are standard deviations of three repetitions. Means with different letters within the same day are significantly different (one-way ANOVA *p* < 0.05; factor: storage temperature (°C)).

Samples were flushed with oxygen and stored at 50, 60, and 70 °C. As expected, high temperatures resulted in faster oxidation. At 70 °C, the sum of these aldehydes (area responses) went approximately two times higher than that at 60 °C (Figure 2). As at 50 °C, no significant changes were seen. These values were in the range of µmol/kg sample. High storage temperature increased the production of volatiles in infant milk formula. Similar results were observed in a study on infant milk formula in powder form [18]. Temperature not only increases the rate of hydroperoxide decomposition but also induces changes in the repartition (percentage, *cis*/*trans* ratio) of the resulting degradation products [11].

**Figure 3:**
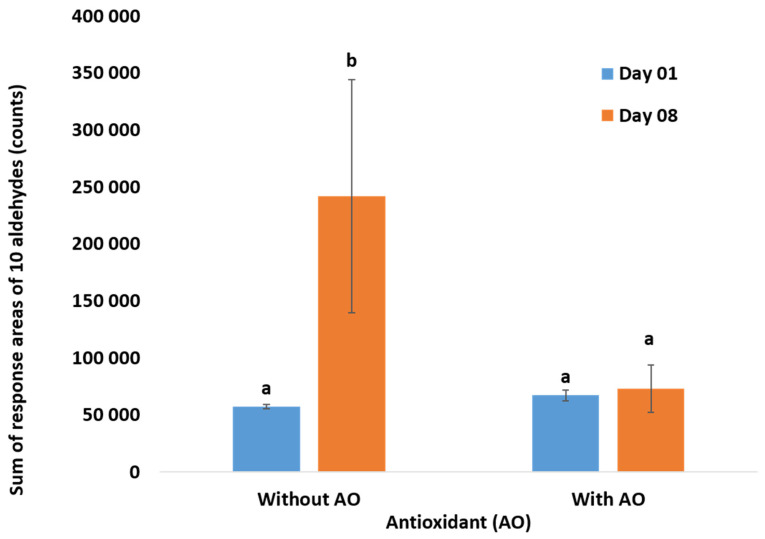
Impact of citric acid, lecithin, and ascorbyl palmitate on the sum of volatiles (responses area) determined by SPME-GC-MS in a liquid infant milk formula. AO means antioxidants and corresponds to the mixture of citric acid, ascorbyl palmitate (E304), and lecithin (use to present an antioxidant activity). The emulsion is stabilized by milk protein. Infant milk formula with antioxidants and without antioxidant (no additives) was stored for 8 days at 70 °C. Values are means and errors bars are standard deviation of three repetitions. Means with different letters are significantly different (one-way ANOVA *p* < 0.05; factor: storage time (days)).

Two preparations of infant milk formulas were studied. The first recipe (No-AOx) did not contain antioxidants. As for the second one (AOx), a mixture of ascorbyl palmitate (E304), citric acid (E330), and lecithin (E322) as a surfactant was used. Figure 3 shows a comparison between these two formulations after storage at 70 °C for 8 days. Even at 70 °C, the antioxidants mixture had a preventive effect. The total sum of volatiles remained stable after 8 days.

The selected antioxidant mixture proved its efficiency even at elevated temperatures. Though lecithin is not classified as a metal ions chelator, or a radical scavenger, it is known for its antioxidant activity. Lecithin’s antioxidant activity can be related to its synergy with other antioxidants (e.g., tocopherols) [54,55,56]. Citric acid chelates iron and retards lipid oxidation [57]. Ascorbyl palmitate has the ability to react with oxygen, scavenge radicals (excited dioxygen, O_2_ e.g., or two ROO), and act in synergy with other antioxidants, for example, by regenerating tocopherol radicals in tocopherols [11,58]. In addition, the added mixture not only combined different antioxidant strategies but also had different sites of action: citric acid in the aqueous phase, ascorbyl palmitate in the oil phase, and lecithin at the interface and in the aqueous phase (in micelle state when its concentration is over the critical micelle concentration (CMC)). In conclusion, when the antioxidant mixture was added, slight changes were occurring in the volatiles’ content even at high temperature. These results confirmed that the variations identified during storage are mainly related to lipid oxidation and not to protein’s reaction with reducing sugars (Maillard reactions) or sugar crystallization.

### 3.2. Infrared Spectroscopy in Mid-Region ATR-FTIR Applied to Infant Milk Formulas

#### 3.2.1. Spectral Changes in Infant Milk Formulas

**Figure 4:**
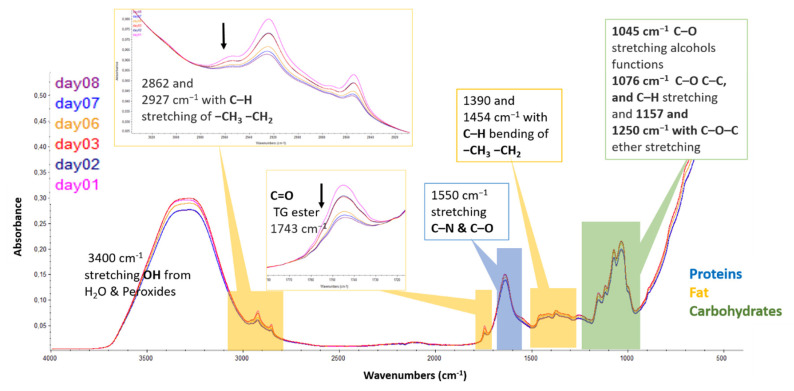
Variation of attenuated total reflectance Fourier-transform infrared (ATR-FTIR) spectra (4000–400 cm^−1^) of liquid infant milk formula without antioxidants stored in dark at 70 °C for 8 days.

The FTIR spectra of infant milk formulas were recorded in the range of 4000–400 cm^−1^. The same absorbance bands were observed for both formulations under different storage conditions. However, changes were noticed after one and six days of storage for samples without and with antioxidants, respectively (Supplementary Materials Figure S3). Attenuation of the bands was more pronounced at higher temperature (70 °C). Figure 4 shows an overlay of the ATR-FTIR spectra of the No-AOx infant formula stored in dark at 70 °C. Several constituents (proteins, carbohydrates, and fats) of infant formula were absorbing in mid-infrared region.

Spectra are the average of three repetitions on three different subsamples. Standard normal variate (SNV) and baseline correction were applied on spectral data.

The first region (3800–2700 cm^−1^) contains the broad water band near 3400 cm^−1^. This band represents the stretching vibrations of the hydroxyl group (OH) from water and hydroperoxides. During storage, the bands at 3012, 2862, and 2927 cm^−1^ decrease, these band are related to fat [59,60]. They represent the stretching vibration of C–H of the *cis* double bond (C=C), CH_3_, and CH_2_, respectively. Once lipids are oxidized, the *cis-*double bond rearranges to form a *trans-*double bond, resulting in the decrease of the band at 3012 cm^−1^ [28,36,61].

The next infrared region (1800–1500 cm^−1^) reveals bands from both protein and fat [59,60]. It contains a band near 1743 cm^−1^ that was attributed to the stretching vibrations of the carbonyl group of triglyceride esters (-C=O) [28,48,61]. The intensity of this band was decreasing during the storage. In the same region, a strong band at 1644 cm^−1^ can be identified as C–H bending vibrations of methylene, overlapping with the O–H deformation of water present in the system. It may also contain the symmetrical stretching vibrations of conjugated C=C at 1634 cm^−1^ [36]. This region covers peaks around 1658 and 1544 cm^−1^, which stand for the vibrations of amide I and II of protein [41,59].

The fingerprint region in 1500–900 cm^−1^ enclosed the CH_2_ bending band at 1452 cm^−1^, and the C–O/CH_2_ stretching and bending band at 1161 cm^−1^ [36,48,59]. The intensity of these bands increased during oxidation because of the decomposition of hydroperoxides through the β-scission mechanism [11,36]. The band at 1377 cm^−1^ was identified as C–H bending vibrations of methyl groups. The area between 1250 and 800 cm^−1^ represents characteristic peaks of various C–O vibrations in carbohydrates [59,60]. Another important band is the one at 968 cm^−1^, which corresponds to the C–H out-of-plane deformation vibration of *trans*-double bonds. Its frequency increases during oxidation (*cis*-double bonds of unsaturated fatty acids undergo isomerization to the *trans*-form) [28,48,62].

#### 3.2.2. Prediction of the Oxidation Levels of Infant Milk Formula (IMF) using ATR-FTIR

Infant milk formulas were kept in the dark at 50, 60, and 70 °C. Through this work, 100 samples were analyzed from both preparations (no additives (No-AOx) and AOx with the ternary mixture of antioxidants). For some samples, the volatile formation was below the GC-MS detection threshold. These samples were disregarded, and a total of sixty-three samples was included in the PLSR method. Samples were divided randomly into a calibration set of 52 samples and a validation set of 11 samples. The volatile content ranged from 43,634 to 188,407 counts. However, a few of the samples showed high values for volatile compounds. This is likely due to the long study duration of 8 days at high temperature (70 °C). Samples also showed minor changes in viscosity and color, which may reflect other phenomena occurring in the same time. The statistic model correlates the sum of volatiles released during storage as measured by GC-MS and predicted from the ATR-FTIR spectral changes in the range of 4000 to 600 cm^−1^. The coefficients of determination (Corr. Coeff), root mean square errors of calibration (RMSEC) and prediction (RMSEP), and the number of factors used and other parameters are summarized in Table 1.

**Figure 5:**
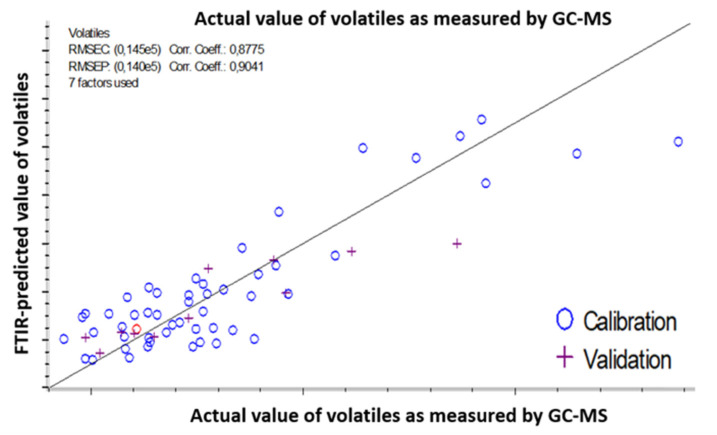
Partial least squares regression (PLSR) analysis prediction model of the oxidation level of liquid milk infant formula: correlation between the actual value of volatiles as measured by headspace solid-phase microextraction gas chromatography coupled to mass spectrometry (HS-SPME-GC-MS) (*x*-axis) and the volatiles values predicted from the spectral changes happening over the whole spectral range of ATR-FTIR spectra (*y*_axis).

Seven factors were selected for this model (respecting the lower error of calibration) (Figure 5). The correlation coefficients were 0.8775 and 0.904 for calibration and prediction, respectively. The errors of calibration (RMSEC) and prediction (RMSEP) were 18% and 17.5%.

Samples were a mixture of infant milk formula with and without the added antioxidant mixture (lecithin, citric acid, and ascorbyl palmitate) at different days (1 to 8) and temperature (50, 60, and 70 °C) of storage.

The ATR-FTIR model is suitable for quality control: the ratio of volatile content range to the error of prediction is superior to 10. These findings proved that the quality of the prediction model is strictly related to the ability of standard methods to accurately monitor lipid oxidation.

### 3.3. Infrared Spectroscopy in the Near-Region (NIRS) Applied on Infant Milk Formulas

#### 3.3.1. Spectral Changes of Infant Milk Formulas

**Figure 6:**
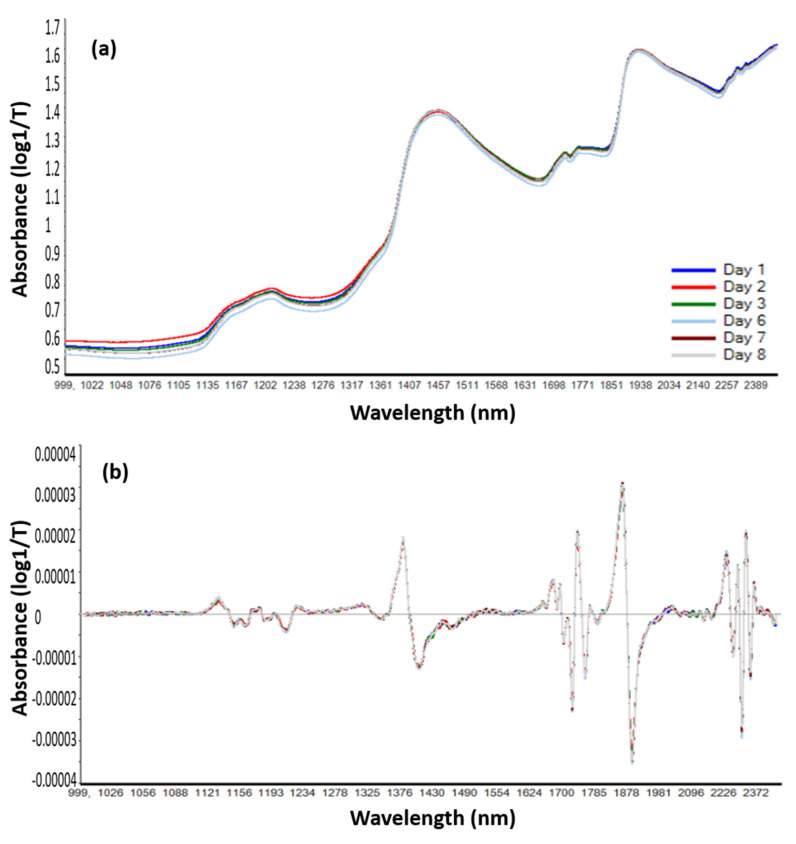
Variation of near infrared spectra of liquid infant milk formula; (**a**) raw, (**b**) first derivative of the NIR signal.

The NIR spectra of IMF were recorded in the range of 1000 to 2500 nm. Derivation allowed the revealing of other bands in addition to the two broad bands of water at 1450 and 1940 nm (Figure 6). The spectra were divided into five ranges. The main peaks and their attribution are summarized in Table 2.

The first part of the NIR spectrum (1120 to 1280 nm) represents the second overtone of C–H from CH_3_, CH_2_, and CH. The second region (1300–1600 nm) combines the first overtone of combinations C–H for CH_3_, CH_2_, and CH, and the first overtone of O–H (H_2_O water) and N–H (CONHR proteins). The third region (1630–1820 nm) represents the first overtone of C–H for CH_3_, CH_2_, and CH. The part of spectra from 1820 to 2180 nm combines the second overtone of C=O and combinations bands of O–H. The last part (2180–2500 nm) represents the combinations of NH–OH and combinations from CH–CC and CC–CC.

Samples without antioxidants were stored in the dark at 70 °C. Samples were analyzed during 8 days. More variations were detected after the application of a second gap (23 points) segment (19 points) derivation.

#### 3.3.2. Prediction of the Oxidation Level of IMF using NIRS

Infant milk formulas were kept in the dark at 50, 60, and 70 °C. Similarly, all samples with total volatiles content below the GC-MS detection limit were excluded and sixty samples were kept for the PLSR model. Samples were once again, divided randomly into calibration and validation sets with 50 and 10 samples, respectively. The sum of volatiles ranged from 43,634 to 142,015 counts, with a mean value of 71,410 counts. The entire spectral range was used to create the prediction model. The parameters used to prepare the NIR prediction model are presented in Table 1.

**Figure 7:**
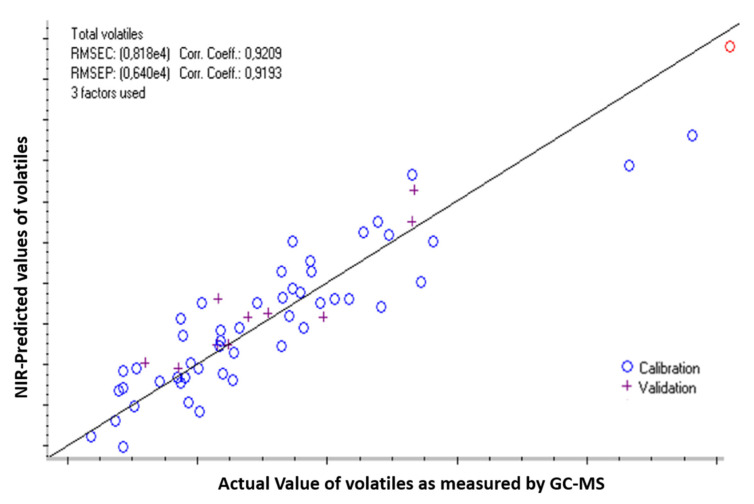
PLSR prediction model for lipid oxidation of infant milk formula using near-infrared spectroscopy (NIR).

Three factors were selected for this model. The low number of factors increases the robustness of the model. The scatter plot of predicted (from NIRS) and measured values of the sum of volatiles is presented in Figure 7.

The scatter plot presents in the *x*-axis the value of the sum of volatiles formed during the storage of liquid infant milk formula (fat oxidation measured by SPME-GC-MS) to the calculated values from the spectral changes during storage (NIR-predicted volatiles value) (*y*-axis). Samples used to build this model were a pool of infant milk formulas with and without the added antioxidant mixture, at different days and temperature of storage.

The characteristics of the NIRS-prediction model are detailed in Table 1. The coefficients of determination (R^2^) of calibration and prediction were 0.921 and 0.919, respectively. The errors of calibration and prediction were 8180 counts (11.4%) and 6400 counts (9%), respectively. The ratio of the error of prediction (RMSEP) to the error of calibration (RMSEC) was 0.78 (<1.2). Since the ratio of data range to the error of prediction was 15.3 (>15). Consequently, this model could be used for quantification purposes [50].

The prediction models developed in this study, were designed to detect the lipid oxidation in infant milk. To the authors’ knowledge, this is the first time, that infrared spectroscopy is used for oxidation monitoring in liquid infant milk formulas. However, multiple regression models can be applied to one recorded infrared spectrum. Therefore, this prediction model can be combined to other product characteristics such as composition, oxidation, and physical proprieties can be simultaneously monitored.

## 4. Conclusions

Infrared spectroscopy could detect lipid oxidation in infant formula containing relatively high levels of unsaturated fats. The ATR-FTIR prediction model may present a useful tool for quality control in emulsion applications. The NIR prediction model may even serve to quantify oxidation in these complex matrices. Infrared spectroscopy has the potential to replace standard techniques, avoiding time-consuming and costly sample preparation. Once the prediction model is calibrated and validated for a given sample and setup, a simple acquisition of infrared spectra is needed to evaluate the oxidation level of samples. Furthermore, novel portable infrared spectrophotometers or devices with narrow optical fibers are now available on the market. ATR-FTIR and NIRS may therefore be implemented in dairy industries for quality control or research and development purposes. Such implementation helps reduce time, effort, and cost. Multiple regression models can be applied to the spectral information, which in addition to the lipid oxidation level, may offer additional insights into other parameters (water, protein, and sugar contents) of the final product. For application developers, these tools offer the ability to rapidly test different mixtures and formulations. To improve the quality and performance of the prediction models, the acceleration conditions should be wisely chosen to ensure measurable oxidation levels but also conserve the physical stability of samples. The viscosity of the studied preparations could influence the lipid oxidation kinetics and volatiles releases. The efficiency of the prediction model is directly related to the accuracy and repeatability of the reference methods. In our case, the conjugated dienes method was unable to quantify low oxidation levels. Analysis of volatiles by SPME-GC-MS may be considered as a very sensitive and efficient method. However, here too optimization is needed, with an appropriate choice of standards and matrix to test. Accordingly, the infrared spectroscopic methods developed herein offer the possibility to predict oxidative processes and to establish optimization strategies for the development of infant formulas.

## Figures and Tables

**Table 1:** Summary of ATR-FTIR and near-infrared (NIR) prediction models used to detect volatiles content in infant milk formula: models’ parameters, number of samples, and results.

Prediction Model	ATR-FTIR	NIRS
Spectral range	4000–600 cm^−1^	800–2400 nm
Pretreatment	Standard normal variate (SNV)–baseline correction	Second derivative (gap 23 points, segment 19 points)
Calibration set (samples)	52	50
Validation set (samples)	11	10
Volatiles content range (counts)	43,634–188,407	43,634–142,015
Number of latent variables	7	3
Coefficient of correlation for calibration (R^2^)	0.877	0.921
Coefficient of correlation for prediction (R^2^)	0.904	0.919
Error of calibration (RMSEC)	14,500 counts (18%)	8180 counts (11.4%)
Error of prediction (RMSEP)	14,000 counts (17.5%)	6400 counts (9%)
RMSEC/RMSEP (<1.2)	0.96	0.78
Volatiles content/RMSEP	10.34	15.37

RMSEC, root mean square errors of calibration; RMSEP, root mean errors of prediction.

**Table 2:** Major bands in NIR spectra of infant milk formula [21,41,45,60].

Range	Wavelength (nm)	Functional Group	Attribution
1	1120–1280	1160/1210 sStretching C–H second overtone of CH_3_ and CH_2_	Fat
2	1280–1600	1440 O–H	Water
		1483 and 1570 N–H first overtone	Protein
		C–H first overtone	Fat
3	1630–1820	1726 and 1765 C–H first overtone for CH_2_ CH	Fat
4	1820–2180	1950–deformation and stretching of O–H	Water
		1992/2110 N–H stretching	Protein
5	2180–2500	2280 N–H/2308 C–H	Protein
		2310/2354 C–H	Fat
		2347 stretching CH_2_ and deformation of =CH_2_ 2264 and 2494	Carbohydrate

## Supplementary Materials

The following are available online at https://www.mdpi.com/2304-8158/9/10/1432/s1, Figure S1: The content of Hexanal (area responses in counts) released from infant milk formula under different storage conditions, Figure S2: The content of 2,4-decadienal (area responses in counts) released from infant milk formula under different storage conditions, Figure S3: Variation of ATR-FTIR spectra of liquid infant milk formula with antioxidant.
