# Supplementary material for: Detection of Lipid Oxidation in Infant Formulas: Application of Infrared Spectroscopy to Complex Food Systems
Source: Foods. 2020 Oct 9;9(10):1432. doi: 10.3390/foods9101432 (PMC7599773; doi:10.3390/foods9101432)
Supplement: Supplementary file 1 [file foods-09-01432-s001.pdf]

Supplementary data

A-Volatiles data of infant milk formulas:

Hexanal was the most abundant aldehyde found in infant milk formulas. Measurement were done using HS-SPME/GC-MS. **Figure 1** and **Figure 2** shows the variations of Hexanal and 2,4-decadienal content in different infant milk preparations stored under different conditions.

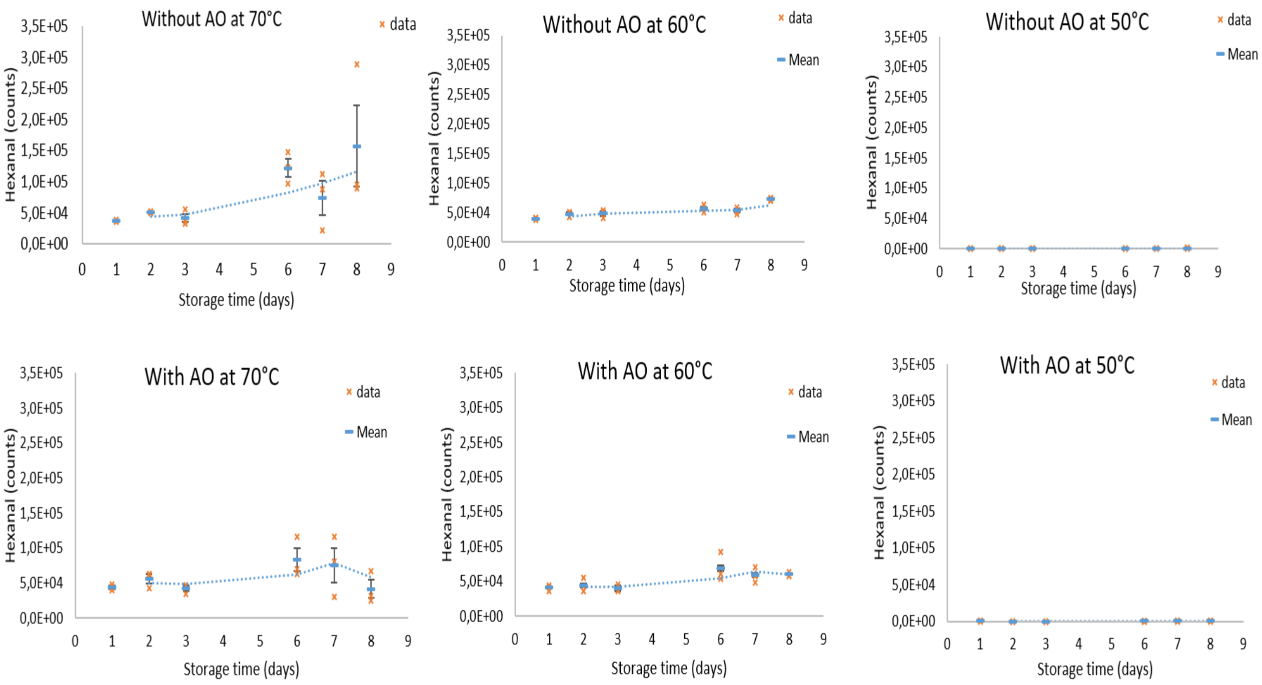

Figure 1. The content of Hexanal (area responses in counts) released from infant milk formula under different storage conditions

Samples were kept in dark at 50, 60 and 70 °C. Two types of preparation's were studied: (with AO): samples with added antioxidant (lecithin, citric acid and ascorbyl palmitate), and (without AO): samples without added antioxidant. Measurement were done on triplicates. Data are presented as mean. Error bars are standard deviations.

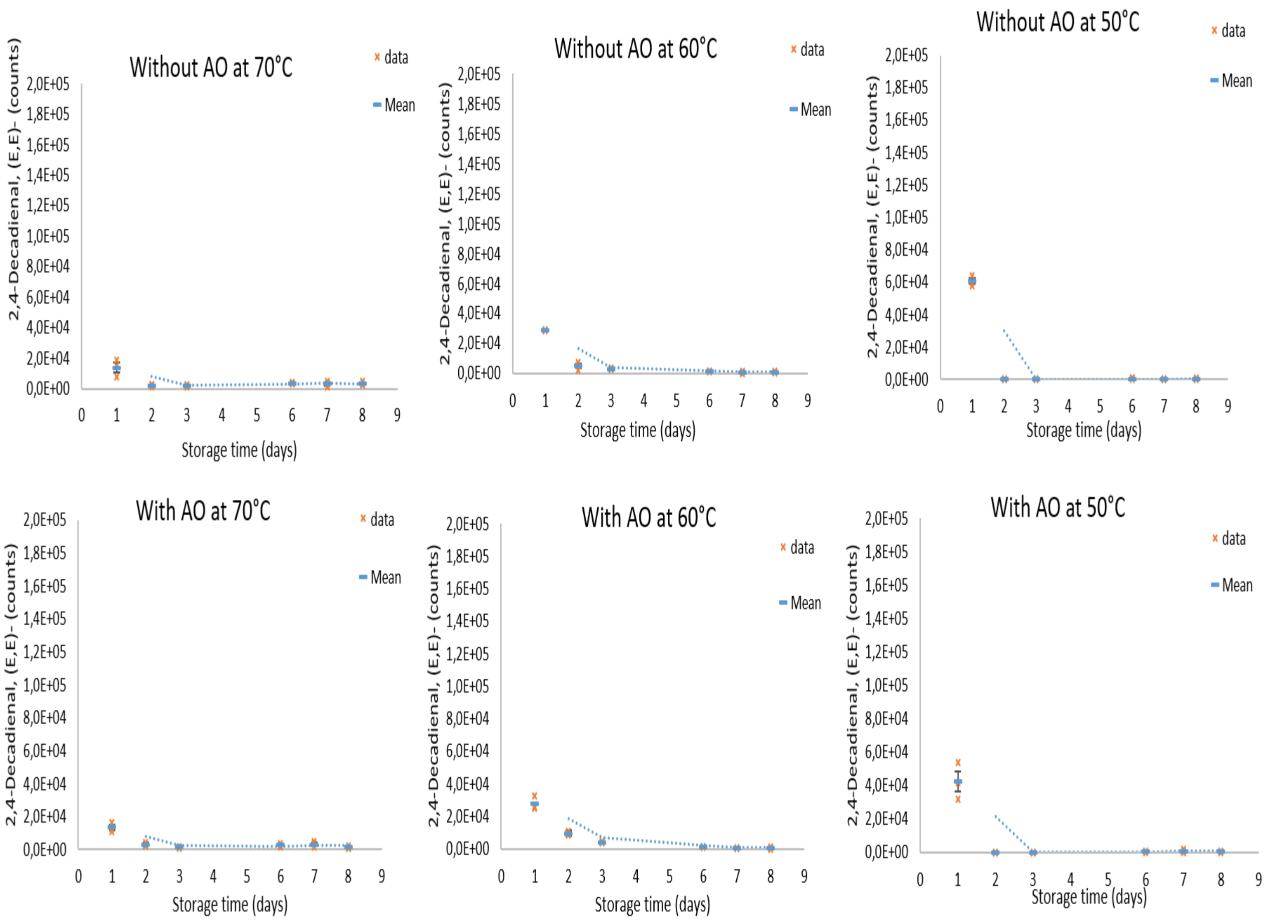

Figure 2. The content of 2,4-decadienal (area responses in counts) released from infant milk formula under different storage conditions

Samples were kept in dark at 50, 60 and 70 °C. Two types of preparation's were studied: (with AO): samples with added antioxidant (lecithin, citric acid and ascorbyl palmitate), and (without AO): samples without added antioxidant. Measurement were done on triplicates. Data are presented as mean. Error bars are standard deviations.

<u>B-ATR-FTIR applied on infant milk formulas:</u>

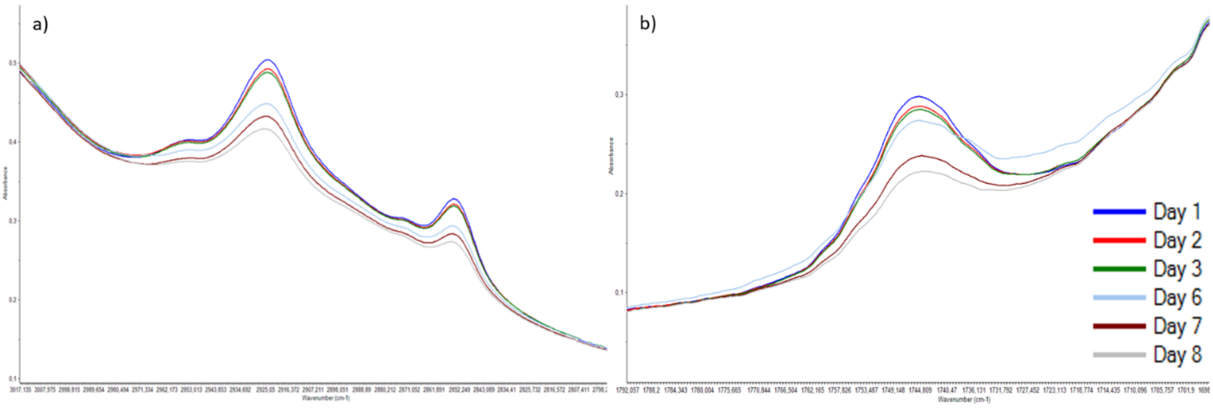

Figure 3. Variation of ATR-FTIR spectra of liquid infant milk formula with antioxidant

The antioxidant mixture contained citric acid, Ascorbyl palmitate and lecithin. Samples were stored in dark at 70 °C for 8 days. Focus on variations in the regions of 3020-2700 cm^{-1}(a) and 1800-1600cm^{-1}(b). Spectra are average of three repetitions on three different subsamples. Standard normal variate (SNV) and baseline correction were applied on spectral data
